# Redetermination of {5-[(7-chloro­quinolinium-4-yl)amino]-2-hy­droxy­benz­yl}diethyl­ammonium dichloride dihydrate

**DOI:** 10.1107/S1600536810031806

**Published:** 2010-08-18

**Authors:** Peter Mangwala Kimpende, Luc Van Meervelt

**Affiliations:** a Katholieke Universiteit Leuven, Department of Chemistry, Celestijnenlaan 200F, B-3001 Leuven (Heverlee), Belgium

## Abstract

The structure of the title compound (common name: amodiaquinium dichloride dihydrate), C_20_H_24_ClN_3_O_2_^+^·2Cl^−^·2H_2_O, was previously determined from powder diffraction data [Llinàs *et al.* (2006 ▶). *Acta Cryst*. E**62**, o4196-o4199]. It has now been refined from diffractometer data to a significantly higher precision. The dihedral angle between the quinoline and benzene rings is 54.57 (6)°. The central amino N atom inter­acts more strongly with the quinoline ring than with the benzene ring, as indicated by the shorter C—N bond length [1.341 (2) Å compared to 1.431 (2) Å]. In the crystal, mol­ecules are packed into a three-dimensional network/supra­molecular structure through hydrogen bonds between the amodiaquinium cations, chloride anions and water mol­ecules.

## Related literature

Amodiaquine, as a dihydro­chloride salt, is often used as a synthetic anti­malarial drug against chloro­quine-sensitive and chloro­quine-resistant strains of *Plasmodium falciparum*, see: Olliaro & Taylor (2003 ▶). For related structures, see: Llinàs *et al.* (2006 ▶); Yennawar & Viswamitra (1991 ▶); Semeniuk *et al.* (2008 ▶).
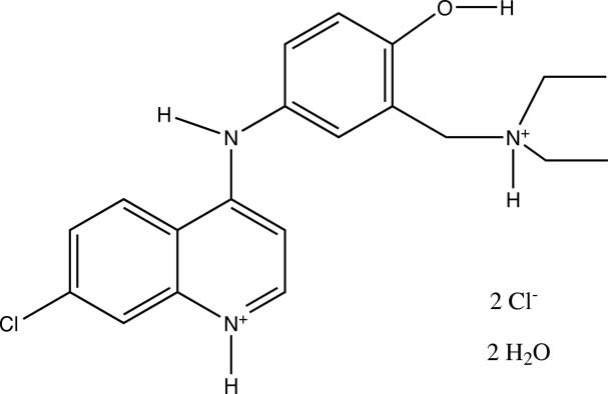


## Experimental

### 

#### Crystal data

C_20_H_24_ClN_3_O^2+^·2Cl^−^·2H_2_O
*M* *_r_* = 464.80
Monoclinic, 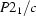

*a* = 7.7622 (1) Å
*b* = 26.8709 (4) Å
*c* = 10.7085 (2) Å
β = 92.784 (1)°
*V* = 2230.91 (6) Å^3^
*Z* = 4
Cu *K*α radiation
μ = 3.94 mm^−1^
*T* = 100 K
0.56 × 0.14 × 0.12 mm

#### Data collection

Bruker SMART 6000 CCD diffractometer
Absorption correction: multi-scan (*SADABS*; Bruker, 1997 ▶) *T*_min_ = 0.312, *T*_max_ = 0.623
31612 measured reflections
3917 independent reflections
3699 reflections with *I* > 2σ(*I*)
*R*_int_ = 0.088

#### Refinement

*R*[*F* ^2^ > 2σ(*F* ^2^)] = 0.038
*wR*(*F* ^2^) = 0.100
*S* = 1.10
3917 reflections
287 parameters
H atoms treated by a mixture of independent and constrained refinement
Δρ_max_ = 0.40 e Å^−3^
Δρ_min_ = −0.32 e Å^−3^

### 

Data collection: *SMART* (Bruker, 1997 ▶); cell refinement: *SAINT* (Bruker, 1997 ▶); data reduction: *SAINT*; program(s) used to solve structure: *SHELXS97* (Sheldrick, 2008 ▶); program(s) used to refine structure: *SHELXL97* (Sheldrick, 2008 ▶); molecular graphics: *PLATON* (Spek, 2009 ▶) and *DIAMOND* (Brandenburg, 2010 ▶); software used to prepare material for publication: *PLATON*.

## Supplementary Material

Crystal structure: contains datablocks I, global. DOI: 10.1107/S1600536810031806/lx2163sup1.cif

Structure factors: contains datablocks I. DOI: 10.1107/S1600536810031806/lx2163Isup2.hkl

Additional supplementary materials:  crystallographic information; 3D view; checkCIF report

## Figures and Tables

**Table 1:** Hydrogen-bond geometry (Å, °)

*D*—H⋯*A*	*D*—H	H⋯*A*	*D*⋯*A*	*D*—H⋯*A*
N1—H1*N*⋯Cl2	0.89 (2)	2.32 (2)	3.1913 (16)	166.8 (19)
N2—H2*N*⋯O2*W*^i^	0.83 (2)	2.07 (2)	2.880 (2)	167 (2)
N3—H3*N*⋯Cl3	0.85 (2)	2.26 (2)	3.0771 (14)	161 (2)
O1—H1*O*⋯Cl2^ii^	0.84	2.22	3.0640 (12)	177
O1*W*—H1*WA*⋯Cl3^iii^	0.88 (3)	2.30 (3)	3.1778 (16)	175 (3)
O1*W*—H1*WB*⋯Cl3^i^	0.80 (3)	2.42 (3)	3.2100 (16)	171 (3)
O2*W*—H2*WA*⋯O1*W*	0.83 (3)	1.95 (3)	2.775 (2)	174 (2)
O2*W*—H2*WB*⋯Cl2^ii^	0.83 (3)	2.33 (3)	3.1585 (15)	173 (3)

Symmetry codes: (i) 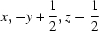; (ii) 
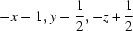
; (iii) 
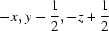
.

**Fig. 1.**
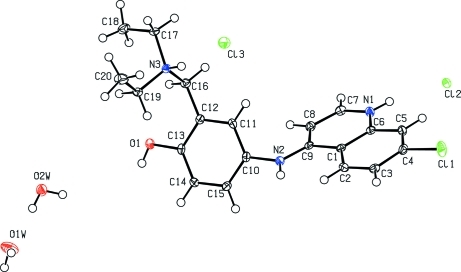
The molecular structure of the title compound with the atom numbering scheme. Displacement ellipsoids are drawn at the 50% probability level. H atoms are presented as a small spheres of arbitrary radius.

**Fig. 2.**
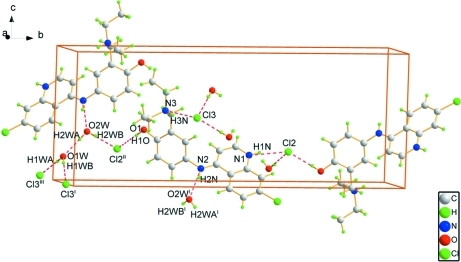
N–H···Cl, N–H···O, O–H···Cl, and O–H···O hydrogen bonds (dotted lines) in the crystal structure of the title compound. [Symmetry codes : (i) x , - y + 1/2, z - 1/2; (ii) -x - 1, y - 1/2, - z + 1/2; (iii) - x , y -1/2, - z + 1/2.]

## supplementary crystallographic information

### Comment

Amodiaquine, 4-[7-chloro-4-quinolinyl)amino]-2-[(diethylamino)methyl]phenol, is as dihydrochloride salt, often used as synthetic antimalarial drug against chloroquine-sensitive and chloroquine-resistant strains of *Plasmodium falciparum* (Olliaro & Taylor, 2003). The single-crystal structure of the monohydrate form has been reported by Yennawar & Viswamitra (1991) and by Semeniuk *et al.* (2008). The room temperature structure of the dihydrate form based on powder diffraction at 1.79 Å resolution has been reported by Llinàs *et al.* (2006). Here we report the crystal structure of the title compound (I) at 100 K and a resolution of 0.84 Å (Fig. 1).

Two N atoms (N1 and N3) are protonated indicating that the dihydrochloride salt of amodiaquine is present. The shape of the molecule is mainly dominated by three torsion angles: C8–C9–N2–C19 (τ_1_ = -7.7 (3)°), C9–N2–C10–C11 (τ_2_ = -52.8 (2)°) and C11–C12–C16–N3 (τ_3_ = -85.85 (18)°). It was suggested by Yennawar & Viswamitra (1991) that the C–N bonds linking both aromatic rings have double-bond character. However, we observe a large difference between both bonds C9–N2 (1.341 (2) Å) and N2–C10 (1.431 (2) Å), indicating that N2 interacts more with the quinoline than with the benzene unit. It is also clear from inspection of τ_1_ and τ_2_ that the overlap of the lone pair of the *sp*^2^-hybridized N2 with the quinoline unit is favoured, and this despite the short H2N···H2 contact distance (2.08 Å). The dihedral angle between the quinoline and benzene units is 54.57 (6)°. An intramolecular close contact between H16A and O1 (2.396 Å) is observed by Llinàs *et al.* (2006). The r.m.s. deviation when fitting the amodiaquinium units obtained by single-crystal and powder diffraction (Llinàs *et al.*, 2006) is 0.0739 Å. The hydrogen bonds in the crystal packing (Table 1, Fig. 2) are similar to those described by Llinàs *et al.* (2006).

### Experimental

Amodiaquinium dichloride dihydrate was purchased from Sigma-Aldrich (Belgium). Colourless crystals were obtained at room temperature by slow evaporation from a DMSO solution of (I).

### Refinement

H atoms of the NH groups and of both waters were located in a difference map. The other H atoms were positioned with idealized geometry using a riding model with C–H = 0.95-0.99 Å. All H atoms were refined with isotropic displacement parameters (set to 1.2 or 1.5 times the *U*_eq_ of the parent atom).

### Crystal data

**Table:**

C_20_H_24_ClN_3_O^2+^·2Cl^−^·2H_2_O	*F*(000) = 976
*M**_r_* = 464.80	*D*_x_ = 1.384 Mg m^−^^3^
Monoclinic, *P*2_1_/*c*	Cu *K*α radiation, λ = 1.54178 Å
Hall symbol: -P 2ybc	Cell parameters from 6662 reflections
*a* = 7.7622 (1) Å	θ = 3.3–70.5°
*b* = 26.8709 (4) Å	µ = 3.94 mm^−^^1^
*c* = 10.7085 (2) Å	*T* = 100 K
β = 92.784 (1)°	Prism, colourless
*V* = 2230.91 (6) Å^3^	0.56 × 0.14 × 0.12 mm
*Z* = 4	

### Data collection

**Table:**

Bruker SMART 6000 CCD diffractometer	3917 independent reflections
Radiation source: fine-focus sealed tube	3699 reflections with *I* > 2σ(*I*)
crossed Göbel mirrors	*R*_int_ = 0.088
Detector resolution: 0.92 pixels mm^-1^	θ_max_ = 66.6°, θ_min_ = 3.3°
ω and φ scans	*h* = −8→9
Absorption correction: multi-scan (*SADABS*; Bruker, 1997)	*k* = −31→31
*T*_min_ = 0.312, *T*_max_ = 0.623	*l* = −12→12
31612 measured reflections	

### Refinement

**Table:**

Refinement on *F*^2^	Primary atom site location: structure-invariant direct methods
Least-squares matrix: full	Secondary atom site location: difference Fourier map
*R*[*F*^2^ > 2σ(*F*^2^)] = 0.038	Hydrogen site location: difference Fourier map
*wR*(*F*^2^) = 0.100	H atoms treated by a mixture of independent and constrained refinement
*S* = 1.10	*w* = 1/[σ^2^(*F*_o_^2^) + (0.0443*P*)^2^ + 1.0601*P*] where *P* = (*F*_o_^2^ + 2*F*_c_^2^)/3
3917 reflections	(Δ/σ)_max_ = 0.001
287 parameters	Δρ_max_ = 0.40 e Å^−^^3^
0 restraints	Δρ_min_ = −0.32 e Å^−^^3^

### Special details

**Table:**

Geometry. All e.s.d.'s (except the e.s.d. in the dihedral angle between two l.s. planes) are estimated using the full covariance matrix. The cell e.s.d.'s are taken into account individually in the estimation of e.s.d.'s in distances, angles and torsion angles; correlations between e.s.d.'s in cell parameters are only used when they are defined by crystal symmetry. An approximate (isotropic) treatment of cell e.s.d.'s is used for estimating e.s.d.'s involving l.s. planes.
Refinement. Refinement of *F*^2^ against ALL reflections. The weighted *R*-factor *wR* and goodness of fit *S* are based on *F*^2^, conventional *R*-factors *R* are based on *F*, with *F* set to zero for negative *F*^2^. The threshold expression of *F*^2^ > σ(*F*^2^) is used only for calculating *R*-factors(gt) *etc*. and is not relevant to the choice of reflections for refinement. *R*-factors based on *F*^2^ are statistically about twice as large as those based on *F*, and *R*- factors based on ALL data will be even larger.

### Fractional atomic coordinates and isotropic or equivalent isotropic displacement parameters (Å^2^)

**Table:**

	*x*	*y*	*z*	*U*_iso_*/*U*_eq_	
C1	−0.2951 (2)	0.51757 (6)	0.06525 (16)	0.0118 (3)	
C2	−0.2063 (2)	0.52058 (6)	−0.04673 (16)	0.0131 (4)	
H2	−0.1539	0.4915	−0.0782	0.016*	
C3	−0.1939 (2)	0.56445 (7)	−0.11087 (16)	0.0140 (4)	
H3	−0.1362	0.5658	−0.1870	0.017*	
C4	−0.2681 (2)	0.60736 (7)	−0.06187 (17)	0.0147 (4)	
C5	−0.3573 (2)	0.60667 (6)	0.04469 (17)	0.0141 (4)	
H5	−0.4080	0.6362	0.0753	0.017*	
C6	−0.3724 (2)	0.56123 (6)	0.10810 (16)	0.0118 (3)	
C7	−0.4831 (2)	0.51792 (7)	0.27924 (16)	0.0141 (4)	
H7	−0.5498	0.5183	0.3513	0.017*	
C8	−0.4081 (2)	0.47445 (6)	0.24440 (16)	0.0138 (4)	
H8	−0.4226	0.4453	0.2928	0.017*	
C9	−0.3097 (2)	0.47234 (6)	0.13779 (16)	0.0118 (3)	
C10	−0.2219 (2)	0.38571 (6)	0.17370 (16)	0.0127 (4)	
C11	−0.1590 (2)	0.38657 (6)	0.29763 (16)	0.0125 (4)	
H11	−0.1216	0.4171	0.3343	0.015*	
C12	−0.1502 (2)	0.34316 (6)	0.36829 (16)	0.0115 (3)	
C13	−0.2084 (2)	0.29835 (6)	0.31382 (17)	0.0121 (3)	
C14	−0.2641 (2)	0.29733 (6)	0.18823 (17)	0.0139 (4)	
H14	−0.2977	0.2667	0.1501	0.017*	
C15	−0.2709 (2)	0.34090 (7)	0.11850 (17)	0.0140 (4)	
H15	−0.3091	0.3400	0.0329	0.017*	
C16	−0.0831 (2)	0.34431 (6)	0.50266 (16)	0.0133 (4)	
H16A	−0.1354	0.3166	0.5485	0.016*	
H16B	−0.1191	0.3759	0.5414	0.016*	
C17	0.1735 (2)	0.34491 (6)	0.65140 (16)	0.0136 (4)	
H17A	0.1177	0.3744	0.6876	0.016*	
H17B	0.2995	0.3509	0.6553	0.016*	
C18	0.1364 (3)	0.29984 (7)	0.73055 (17)	0.0206 (4)	
H18A	0.0146	0.2906	0.7179	0.031*	
H18B	0.1614	0.3077	0.8189	0.031*	
H18C	0.2090	0.2720	0.7061	0.031*	
C19	0.1800 (2)	0.29476 (7)	0.45331 (16)	0.0145 (4)	
H19A	0.1337	0.2938	0.3655	0.017*	
H19B	0.1390	0.2646	0.4960	0.017*	
C20	0.3754 (3)	0.29404 (8)	0.45504 (19)	0.0242 (4)	
H20A	0.4172	0.3254	0.4210	0.036*	
H20B	0.4137	0.2662	0.4040	0.036*	
H20C	0.4217	0.2900	0.5412	0.036*	
N1	−0.4650 (2)	0.56009 (6)	0.21445 (14)	0.0137 (3)	
H1N	−0.514 (3)	0.5883 (9)	0.237 (2)	0.016*	
N2	−0.2317 (2)	0.43058 (5)	0.10177 (14)	0.0126 (3)	
H2N	−0.190 (3)	0.4290 (8)	0.032 (2)	0.015*	
N3	0.1117 (2)	0.33997 (5)	0.51605 (14)	0.0114 (3)	
H3N	0.152 (3)	0.3658 (9)	0.482 (2)	0.015 (5)*	
O1	−0.20278 (17)	0.25706 (4)	0.38692 (12)	0.0160 (3)	
H1O	−0.2483	0.2331	0.3472	0.024*	
O1W	−0.0091 (2)	0.02817 (6)	0.17421 (15)	0.0321 (4)	
H1WA	−0.080 (4)	0.0063 (12)	0.139 (3)	0.038*	
H1WB	0.063 (4)	0.0345 (11)	0.126 (3)	0.038*	
O2W	−0.08987 (19)	0.09265 (5)	0.36517 (13)	0.0201 (3)	
H2WA	−0.073 (3)	0.0731 (10)	0.307 (3)	0.024*	
H2WB	−0.157 (4)	0.1146 (10)	0.337 (2)	0.024*	
Cl1	−0.24427 (7)	0.663720 (16)	−0.13878 (5)	0.02505 (15)	
Cl2	−0.63123 (6)	0.667531 (14)	0.24913 (4)	0.01525 (13)	
Cl3	0.24523 (6)	0.444735 (15)	0.45660 (4)	0.02012 (14)	

### Atomic displacement parameters (Å^2^)

**Table:**

	*U*^11^	*U*^22^	*U*^33^	*U*^12^	*U*^13^	*U*^23^
C1	0.0116 (8)	0.0129 (8)	0.0105 (8)	−0.0019 (7)	−0.0021 (7)	0.0008 (6)
C2	0.0133 (8)	0.0126 (8)	0.0134 (8)	−0.0003 (7)	0.0005 (7)	−0.0025 (6)
C3	0.0148 (9)	0.0163 (8)	0.0110 (8)	−0.0015 (7)	0.0004 (7)	0.0011 (7)
C4	0.0153 (9)	0.0129 (8)	0.0156 (8)	−0.0009 (7)	−0.0013 (7)	0.0041 (7)
C5	0.0144 (9)	0.0110 (8)	0.0168 (8)	0.0017 (7)	0.0000 (7)	−0.0009 (7)
C6	0.0100 (8)	0.0141 (8)	0.0110 (8)	−0.0001 (6)	−0.0012 (7)	−0.0003 (6)
C7	0.0133 (8)	0.0174 (9)	0.0117 (8)	−0.0014 (7)	0.0017 (7)	0.0012 (7)
C8	0.0144 (8)	0.0135 (8)	0.0135 (8)	−0.0013 (7)	0.0002 (7)	0.0023 (6)
C9	0.0109 (8)	0.0127 (8)	0.0115 (8)	−0.0005 (6)	−0.0038 (7)	0.0002 (6)
C10	0.0128 (8)	0.0119 (8)	0.0134 (8)	0.0025 (7)	0.0013 (7)	0.0018 (6)
C11	0.0128 (8)	0.0093 (8)	0.0153 (8)	0.0010 (6)	0.0014 (7)	−0.0012 (6)
C12	0.0104 (8)	0.0134 (8)	0.0108 (8)	0.0020 (6)	0.0024 (7)	0.0006 (6)
C13	0.0098 (8)	0.0106 (8)	0.0159 (9)	0.0018 (6)	0.0015 (7)	0.0030 (6)
C14	0.0154 (9)	0.0106 (8)	0.0154 (9)	−0.0005 (6)	−0.0014 (7)	−0.0011 (6)
C15	0.0145 (9)	0.0152 (9)	0.0120 (8)	0.0023 (7)	−0.0018 (7)	0.0001 (6)
C16	0.0127 (9)	0.0154 (8)	0.0119 (8)	0.0008 (7)	0.0014 (7)	−0.0010 (6)
C17	0.0160 (9)	0.0158 (8)	0.0087 (8)	−0.0004 (7)	−0.0016 (7)	−0.0013 (6)
C18	0.0265 (10)	0.0228 (10)	0.0123 (8)	−0.0023 (8)	−0.0021 (8)	0.0032 (7)
C19	0.0168 (9)	0.0153 (8)	0.0113 (8)	0.0019 (7)	−0.0004 (7)	−0.0029 (6)
C20	0.0173 (10)	0.0341 (11)	0.0210 (10)	0.0059 (8)	−0.0007 (8)	−0.0101 (8)
N1	0.0149 (8)	0.0124 (7)	0.0140 (7)	0.0027 (6)	0.0025 (6)	−0.0007 (6)
N2	0.0169 (8)	0.0113 (7)	0.0098 (7)	0.0003 (6)	0.0021 (6)	0.0013 (5)
N3	0.0137 (8)	0.0113 (7)	0.0093 (7)	−0.0015 (6)	0.0004 (6)	0.0019 (6)
O1	0.0223 (7)	0.0093 (6)	0.0163 (6)	−0.0028 (5)	−0.0011 (5)	0.0030 (5)
O1W	0.0378 (9)	0.0334 (9)	0.0259 (8)	−0.0084 (7)	0.0113 (7)	−0.0119 (7)
O2W	0.0264 (8)	0.0181 (7)	0.0161 (6)	0.0032 (6)	0.0033 (6)	0.0022 (5)
Cl1	0.0353 (3)	0.0141 (2)	0.0266 (3)	0.00221 (18)	0.0109 (2)	0.00951 (17)
Cl2	0.0173 (2)	0.0126 (2)	0.0157 (2)	0.00398 (15)	−0.00045 (17)	−0.00229 (14)
Cl3	0.0266 (3)	0.0168 (2)	0.0171 (2)	−0.00626 (17)	0.00212 (19)	0.00298 (15)

### Geometric parameters (Å, °)

**Table:**

C1—C6	1.405 (2)	C14—H14	0.9500
C1—C2	1.414 (3)	C15—H15	0.9500
C1—C9	1.450 (2)	C16—N3	1.516 (2)
C2—C3	1.370 (3)	C16—H16A	0.9900
C2—H2	0.9500	C16—H16B	0.9900
C3—C4	1.402 (3)	C17—N3	1.510 (2)
C3—H3	0.9500	C17—C18	1.514 (2)
C4—C5	1.364 (3)	C17—H17A	0.9900
C4—Cl1	1.7381 (17)	C17—H17B	0.9900
C5—C6	1.405 (2)	C18—H18A	0.9800
C5—H5	0.9500	C18—H18B	0.9800
C6—N1	1.376 (2)	C18—H18C	0.9800
C7—N1	1.340 (2)	C19—N3	1.497 (2)
C7—C8	1.365 (3)	C19—C20	1.516 (3)
C7—H7	0.9500	C19—H19A	0.9900
C8—C9	1.405 (3)	C19—H19B	0.9900
C8—H8	0.9500	C20—H20A	0.9800
C9—N2	1.340 (2)	C20—H20B	0.9800
C10—C15	1.386 (3)	C20—H20C	0.9800
C10—C11	1.392 (3)	N1—H1N	0.89 (2)
C10—N2	1.431 (2)	N2—H2N	0.83 (3)
C11—C12	1.390 (2)	N3—H3N	0.85 (3)
C11—H11	0.9500	O1—H1O	0.8400
C12—C13	1.403 (2)	O1W—H1WA	0.88 (3)
C12—C16	1.507 (2)	O1W—H1WB	0.80 (3)
C13—O1	1.357 (2)	O2W—H2WA	0.83 (3)
C13—C14	1.393 (3)	O2W—H2WB	0.84 (3)
C14—C15	1.388 (3)		
			
C6—C1—C2	117.57 (16)	C12—C16—N3	112.69 (14)
C6—C1—C9	118.64 (16)	C12—C16—H16A	109.1
C2—C1—C9	123.79 (16)	N3—C16—H16A	109.1
C3—C2—C1	121.54 (16)	C12—C16—H16B	109.1
C3—C2—H2	119.2	N3—C16—H16B	109.1
C1—C2—H2	119.2	H16A—C16—H16B	107.8
C2—C3—C4	118.68 (16)	N3—C17—C18	114.01 (14)
C2—C3—H3	120.7	N3—C17—H17A	108.8
C4—C3—H3	120.7	C18—C17—H17A	108.8
C5—C4—C3	122.46 (16)	N3—C17—H17B	108.8
C5—C4—Cl1	118.58 (14)	C18—C17—H17B	108.8
C3—C4—Cl1	118.96 (14)	H17A—C17—H17B	107.6
C4—C5—C6	118.29 (16)	C17—C18—H18A	109.5
C4—C5—H5	120.9	C17—C18—H18B	109.5
C6—C5—H5	120.9	H18A—C18—H18B	109.5
N1—C6—C1	120.02 (16)	C17—C18—H18C	109.5
N1—C6—C5	118.59 (16)	H18A—C18—H18C	109.5
C1—C6—C5	121.40 (16)	H18B—C18—H18C	109.5
N1—C7—C8	121.68 (16)	N3—C19—C20	112.36 (15)
N1—C7—H7	119.2	N3—C19—H19A	109.1
C8—C7—H7	119.2	C20—C19—H19A	109.1
C7—C8—C9	120.83 (16)	N3—C19—H19B	109.1
C7—C8—H8	119.6	C20—C19—H19B	109.1
C9—C8—H8	119.6	H19A—C19—H19B	107.9
N2—C9—C8	122.57 (16)	C19—C20—H20A	109.5
N2—C9—C1	119.97 (16)	C19—C20—H20B	109.5
C8—C9—C1	117.46 (16)	H20A—C20—H20B	109.5
C15—C10—C11	119.80 (16)	C19—C20—H20C	109.5
C15—C10—N2	119.74 (15)	H20A—C20—H20C	109.5
C11—C10—N2	120.43 (15)	H20B—C20—H20C	109.5
C12—C11—C10	120.72 (16)	C7—N1—C6	121.31 (15)
C12—C11—H11	119.6	C7—N1—H1N	121.8 (14)
C10—C11—H11	119.6	C6—N1—H1N	116.8 (14)
C11—C12—C13	119.18 (16)	C9—N2—C10	124.30 (16)
C11—C12—C16	120.54 (16)	C9—N2—H2N	120.2 (15)
C13—C12—C16	120.26 (15)	C10—N2—H2N	115.4 (15)
O1—C13—C14	122.72 (15)	C19—N3—C17	113.52 (13)
O1—C13—C12	117.46 (16)	C19—N3—C16	113.13 (14)
C14—C13—C12	119.79 (16)	C17—N3—C16	110.65 (14)
C15—C14—C13	120.30 (16)	C19—N3—H3N	108.9 (15)
C15—C14—H14	119.8	C17—N3—H3N	103.8 (15)
C13—C14—H14	119.8	C16—N3—H3N	106.1 (15)
C10—C15—C14	120.05 (16)	C13—O1—H1O	109.5
C10—C15—H15	120.0	H1WA—O1W—H1WB	108 (3)
C14—C15—H15	120.0	H2WA—O2W—H2WB	107 (2)
			
C6—C1—C2—C3	0.7 (3)	C11—C12—C13—O1	−177.99 (16)
C9—C1—C2—C3	−178.97 (16)	C16—C12—C13—O1	0.6 (2)
C1—C2—C3—C4	1.5 (3)	C11—C12—C13—C14	3.8 (3)
C2—C3—C4—C5	−2.5 (3)	C16—C12—C13—C14	−177.60 (16)
C2—C3—C4—Cl1	177.02 (14)	O1—C13—C14—C15	178.55 (16)
C3—C4—C5—C6	1.1 (3)	C12—C13—C14—C15	−3.4 (3)
Cl1—C4—C5—C6	−178.43 (13)	C11—C10—C15—C14	2.8 (3)
C2—C1—C6—N1	178.00 (16)	N2—C10—C15—C14	−179.04 (17)
C9—C1—C6—N1	−2.3 (2)	C13—C14—C15—C10	0.1 (3)
C2—C1—C6—C5	−2.1 (3)	C11—C12—C16—N3	−85.8 (2)
C9—C1—C6—C5	177.54 (16)	C13—C12—C16—N3	95.62 (19)
C4—C5—C6—N1	−178.85 (16)	C8—C7—N1—C6	1.3 (3)
C4—C5—C6—C1	1.3 (3)	C1—C6—N1—C7	0.2 (3)
N1—C7—C8—C9	−0.6 (3)	C5—C6—N1—C7	−179.66 (16)
C7—C8—C9—N2	178.87 (17)	C8—C9—N2—C10	−7.7 (3)
C7—C8—C9—C1	−1.6 (3)	C1—C9—N2—C10	172.72 (15)
C6—C1—C9—N2	−177.47 (16)	C15—C10—N2—C9	129.05 (19)
C2—C1—C9—N2	2.2 (3)	C11—C10—N2—C9	−52.8 (3)
C6—C1—C9—C8	2.9 (2)	C20—C19—N3—C17	−59.3 (2)
C2—C1—C9—C8	−177.40 (17)	C20—C19—N3—C16	173.55 (15)
C15—C10—C11—C12	−2.3 (3)	C18—C17—N3—C19	−55.1 (2)
N2—C10—C11—C12	179.55 (16)	C18—C17—N3—C16	73.33 (19)
C10—C11—C12—C13	−1.0 (3)	C12—C16—N3—C19	−55.29 (19)
C10—C11—C12—C16	−179.61 (16)	C12—C16—N3—C17	176.05 (14)

### Hydrogen-bond geometry (Å, °)

**Table:**

*D*—H···*A*	*D*—H	H···*A*	*D*···*A*	*D*—H···*A*
N1—H1N···Cl2	0.89 (2)	2.32 (2)	3.1913 (16)	166.8 (19)
N2—H2N···O2W^i^	0.83 (2)	2.07 (2)	2.880 (2)	167 (2)
N3—H3N···Cl3	0.85 (2)	2.26 (2)	3.0771 (14)	161 (2)
O1—H1O···Cl2^ii^	0.84	2.22	3.0640 (12)	177
O1W—H1WA···Cl3^iii^	0.88 (3)	2.30 (3)	3.1778 (16)	175 (3)
O1W—H1WB···Cl3^i^	0.80 (3)	2.42 (3)	3.2100 (16)	171 (3)
O2W—H2WA···O1W	0.83 (3)	1.95 (3)	2.775 (2)	174 (2)
O2W—H2WB···Cl2^ii^	0.83 (3)	2.33 (3)	3.1585 (15)	173 (3)

Symmetry codes: (i) *x*, −*y*+1/2, *z*−1/2; (ii) −*x*−1, *y*−1/2, −*z*+1/2; (iii) −*x*, *y*−1/2, −*z*+1/2.

## References

[bb1] Brandenburg, K. (2010). *DIAMOND* Crystal Impact GbR, Bonn, Germany.

[bb2] Bruker (1997). *SMART*, *SAINT* and *SADABS* Bruker AXS Inc., Madison, Wisconsin, USA.

[bb3] Llinàs, A., Fábián, L., Burley, J. C., van de Streek, J. & Goodman, J. M. (2006). *Acta Cryst.* E**62**, o4196–o4199.

[bb4] Olliaro, P. L. & Taylor, W. R. J. (2003). *J. Exp. Biol.* **206**, 3753–3759. 10.1242/jeb.00653 14506210

[bb5] Semeniuk, A., Niedospial, A., Kalinowska-Tluscik, J., Nitek, W. & Oleksyn, B. J. (2008). *J. Mol. Struct.* **875**, 32–41.

[bb6] Sheldrick, G. M. (2008). *Acta Cryst.* A**64**, 112–122. 10.1107/S0108767307043930 18156677

[bb7] Spek, A. L. (2009). *Acta Cryst.* D**65**, 148–155. 10.1107/S090744490804362X PMC2631630 19171970

[bb8] Yennawar, H. P. & Viswamitra, M. A. (1991). *Curr. Sci.* **61**, 39–43.

